# Characterization of Antidepressant Consumption in a Portuguese Inland Population

**DOI:** 10.3390/healthcare13172177

**Published:** 2025-08-31

**Authors:** Sofia Soares, Tiago Rosado, Vítor Hugo Santos, Cristina Rei, Patricia Amantegui, António Pissarra da Costa, Telma Chaves, Rita Valente, Fábio Duarte, Susana Pacheco, Marco Martins, Kátia Dias, Patricia Costa, Rui Costa, Sílvia Castro, Diana Sousa, Diana Figueiredo, Isabel Soares, Salomé Mouta, Bianca Jesus, Ana Pires, Cândida Ribeiro, Sónia Lobo, Leonor Correia, Sofia Malés, Fátima Vale, Carina Moita, Carolina Moura, Joana Sousa, Luís Rafael Afonso, Rita Santinho Costa, Mário Barroso, Eugenia Gallardo

**Affiliations:** 1 RISE-Health, Departamento de Ciências Médicas, Faculdade de Ciências da Saúde, Universidade da Beira Interior, Avenida Infante D. Henrique, 6200-506 Covilhã, Portugal; sofia_soares_26@hotmail.com (S.S.); vitorhj7@gmail.com (V.H.S.); 2 Laboratório de Fármaco-Toxicologia—Ubimedical, Universidade da Beira Interior, Estrada Municipal 506, 6200-284 Covilhã, Portugal; 3 Centro Académico Clínico das Beiras (CACB)—Missão de Problemas Relacionados com Toxicofilias, Avenida Infante D. Henrique, 6200-506 Covilhã, Portugal; 4 Departamento de Psiquiatria e Saúde Mental, Unidade Local de Saúde Cova da Beira, Alameda Pêro da Covilhã, 6200-251 Covilhã, Portugal; 5 Serviço de Patologia Clínica, Unidade Local de Saúde Cova da Beira, Alameda Pêro da Covilhã, 6200-251 Covilhã, Portugal; cristinarei@chcbeira.min-saude.pt (C.R.); pibarzabal@chcbeira.min-saude.pt (P.A.); 6 Irmãs Hospitaleiras Guarda—Casa de Saúde Bento Menni, Rua José dos Santos no. 40, Bairro da Luz, 6300-575 Guarda, Portugal; dir.clinica.csbm@irmashospitaleiras.pt (A.P.d.C.); telma.marisaferreira@gmail.com (T.C.); rita.valente1@gmail.com (R.V.); duartefabio20@hotmail.com (F.D.); su.enf.guarda@gmail.com (S.P.); dir.enf.csbm@irmashospitaleiras.pt (M.M.); katia.dias91@hotmail.com (K.D.); patriciacosta@outlook.pt (P.C.); ruipmcosta@gmail.com (R.C.); 7 Departamento de Psiquiatria e Saúde Mental, Unidade Local de Saúde da Guarda, Avenida Rainha Dona Amélia, 6300-858 Guarda, Portugal; silvia.castro@ulsguarda.min-saude.pt (S.C.); diana.sousa@ulsguarda.min-saude.pt (D.S.); diana.figueiredo@ulsguarda.min-saude.pt (D.F.); mafalda.soares@ulsguarda.min-saude.pt (I.S.); candida.pinto@ulsguarda.min-saude.pt (S.M.); bianca.jesus@ulsguarda.min-saude.pt (B.J.); ana.c.pires@ulsguarda.min-saude.pt (A.P.); candidadomingues@ulsguarda.min-saude.pt (C.R.); sonia.lobo@ulsguarda.min-saude.pt (S.L.); leonor.correia@ulsguarda.min-saude.pt (L.C.); malessofia36@gmail.com (S.M.); 8 Serviço de Patologia Clínica, Unidade Local de Saúde da Guarda, Avenida Rainha Dona Amélia, 6300-858 Guarda, Portugal; fatima.vale@ulsguarda.min-saude.pt (F.V.); carina.moita@ulsguarda.min-saude.pt (C.M.); carolinamoura@msn.com (C.M.); joana.sousa@ulsguarda.min-saude.pt (J.S.); 9 Biotechnology Research, Innovation and Design for Health Products, Instituto Politécnico da Guarda (BRIDGE-IPG), Avenida Dr. Francisco Sá Carneiro no. 50, 6300-559 Guarda, Portugal; 10 Unidade Local de Saúde Cova da Beira—Unidade de Saúde Familiar Herminius, Alameda Pêro da Covilhã No. 2, 6200-507 Covilhã, Portugal; lrafonso.mgf@gmail.com (L.R.A.); 003444@arscentro.min-saude.pt (R.S.C.); 11 Instituto Nacional de Medicina Legal e Ciências Forenses, Delegação do Sul, Serviço de Química e Toxicologia Forenses, Rua Manuel Bento de Sousa no. 3, 1169-201 Lisboa, Portugal; mbarroso@alphabiolabs.com; 12 AlphaBiolabs, 14 Webster Court, Carina Park, Warrington WA5 8WD, UK

**Keywords:** antidepressants consumption, Portuguese population, surveys, characterization, statistical data

## Abstract

**Background/Objectives**: Mental disorders are a growing global concern, with depression being among the most prevalent. Portugal ranks second in antidepressant consumption within the OECD, following a threefold increase between 2000 and 2020. In inland regions such as Beira Interior, reduced healthcare services and distance from major hospitals further complicate access to care. This study analysed 142 patients from Beira Interior undergoing antidepressant therapy to characterise their demographic and clinical profile and to assess associations with adverse effects. **Methods:** A cross-sectional survey collected demographic data, clinical diagnoses, prescribed antidepressants, concomitant medications, and reported adverse effects. Both descriptive and inferential statistical analyses were performed. **Results:** Most participants were female (81.0%), with a mean age of 57.8 years. Major depression was the most common diagnosis (76.1%). Selective serotonin reuptake inhibitors (47.4%) and trazodone (27.8%) were the most prescribed agents. Treatment had lasted one to five years in 59.9% of cases. Concomitant use of benzodiazepines (76.8%) and antipsychotics (48.6%) was frequent. Reported adverse effects included anticholinergic symptoms (38.7%) and confusion/agitation (26.8%). Women were more likely to use serotonin modulators, while patients >64 years had higher odds of using tetracyclic/unicyclic antidepressants, serotonin modulators, and multiple antidepressants. These classes were significantly associated with increased adverse effects. **Conclusions:** The findings reveal important risks related to polypragmasia and adverse reactions, underscoring the need for individualised prescribing, rigorous monitoring, and strict adherence to guidelines. Larger, stratified, and longitudinal studies are needed to clarify causality and optimise treatment outcomes.

## 1. Introduction

Mental health disorders are a growing global concern, with depression among the most prevalent conditions. Portugal is one of the most affected countries in Europe [1,2,3,4,5]. The rising incidence of these disorders is having a significant impact on public health and increasing the demand for effective treatments [6,7,8]. Antidepressants have therefore become the most widely prescribed class of medication, due to their proven efficacy in reducing symptoms and improving patients’ well-being [8,9,10].

In recent years, antidepressant use has risen considerably, particularly during the COVID-19 pandemic [11,12,13,14]. Portugal ranks second among the Organisation for Economic Cooperation and Development (OECD) countries in antidepressant consumption, with a threefold increase between 2000 and 2020 [15]. According to the Autoridade Nacional do Medicamento e Produtos de Saúde, I.P. (INFARMED), between 2017 and 2023, the number of prescribed and subsidised antidepressants dispensed in community pharmacies in mainland Portugal increased by 44%, totalling around 3.5 million packages. Detailing by class of antidepressants for the same timeframe, there was a 6% increase in When examined by pharmacological class, distinct patterns were observed. Tricyclic antidepressants (TCAs) rose by 6%, with amitriptyline prescriptions increasing by 13% and clomipramine decreasing by 13%. Tetracyclic (TeCAs) and unicyclic antidepressants increased by 57%, particularly bupropion (+94%) and mirtazapine (+49%), while maprotiline fell by 37%. Selective serotonin reuptake inhibitors (SSRIs) rose by 35%, with sertraline (+53%), citalopram/escitalopram (+45%), fluoxetine (+14%), fluvoxamine (+14%), and paroxetine (+4%). Serotonin–norepinephrine reuptake inhibitors (SNRIs) increased by 58%, with duloxetine prescriptions doubling (+100%) and venlafaxine rising by 45%. Finally, serotonin modulators (5-HT modulators), represented only by trazodone, increased by 63% [16].

Beyond these national trends, regional disparities remain a major concern. In Portugal’s inland regions, including Beira Interior, the closure of local health services and the distance to major hospitals have created one of the lowest levels of geographical access to healthcare in the country [17]. Characterizing antidepressant consumption in the Beira Interior region is crucial for understanding regional disparities in mental health care access and needs. Additional aspects, such as geographic and social isolation (rural areas), low and middle-income, the aging of the population, polypragmasia, low funding for mental health treatment, and the stigma associated with seeking psychological help, make this population relevant for the study of antidepressant consumption in Portugal [4,5,17,18,19,20]. This study was conducted in the Beira Interior region to profile antidepressant users and identify patterns of use. Surveys were applied to institutionalised patients (46% of participants) and outpatients. The analysis focused on demographic and clinical data related to antidepressant therapy, particularly sex, age, and reported adverse effects. Comprehensive statistical methods were applied to explore associations between these variables and provide a clearer understanding of antidepressant treatment in this regional context.

## 2. Materials and Methods

This cross-sectional observational study was conducted in the Beira Interior region of Portugal between early 2022 and late 2023, depending on the institution’s recruitment period, and included a total of 142 participants. Three healthcare facilities participated: Casa de Saúde Bento Menni—Irmãs Hospitaleiras Guarda, Unidade Local de Saúde da Guarda, and Centro Hospitalar Universitário Cova da Beira. These institutions, among the main referral centres for psychiatric and mental health care in the region, were selected for their representativeness and because they agreed to collaborate. They provide care for both institutionalised psychiatric patients and outpatients, ensuring the inclusion of individuals with diverse clinical profiles and treatment contexts.

The study population consisted of adult patients under psychiatric follow-up at the participating centres during the study period. A non-probability sampling strategy was applied, using a frame of patients actively receiving psychiatric care. Recruitment began with pre-screening by medical and nursing teams, who reviewed electronic and paper medical records to verify eligibility. Eligible participants were approached directly: outpatients during scheduled consultations and inpatients during routine clinical rounds. All individuals meeting the criteria were consecutively invited, ensuring that no eligible patient was systematically excluded. The study purpose, objectives, and procedures were explained, and written informed consent was obtained from each participant or their legal representative.

Regarding inclusion criteria, participants were required to meet the following conditions: age 18 years or older, a confirmed diagnosis of depressive disorder by a psychiatrist according to DSM-5 criteria, current treatment with antidepressants prescribed by a specialist, and voluntary agreement to participate in the survey, provided either by the patient or their legal representative. Potential participants for this study were excluded if they did not meet at least one of the inclusion criteria described above.

Data was collected using a structured questionnaire specifically designed for this study. Variables included demographic characteristics (age, sex), primary psychiatric diagnosis, antidepressant medication, treatment duration, concomitant pharmacological therapies, general physical health, other psychiatric comorbidities, and adverse effects observed or reported. The instrument was developed from the study protocol and reviewed by psychiatric healthcare professionals for clarity and relevance. As it was designed solely to record objective, verifiable data rather than latent constructs, formal psychometric validation was not applicable. A sample of the survey instrument is provided as Supplementary Material. The final version was administered in person by trained staff and did not include any personally identifiable information, thereby ensuring anonymity and confidentiality.

The study followed the principles of the Declaration of Helsinki. Ethical approval was obtained from the Ethics Committees of all participating institutions: Casa de Saúde Bento Menni—Irmãs Hospitaleiras Guarda (Report 3/2022, 29 December 2022), Unidade Local de Saúde da Guarda (Report 41/2023, 6 October 2023), and Centro Hospitalar Universitário Cova da Beira (Approval 07/2023, 14 March 2023).

Data analysis was conducted using IBM SPSS Statistics (version 27). Descriptive statistics, including frequencies and percentages, were used to characterise sociodemographic and clinical variables such as age, sex, diagnosis, antidepressant use, treatment duration, concomitant medications, physical health, and adverse effects. Associations between variables were assessed using Pearson’s chi-squared (χ^2^) test or Fisher’s exact test. Pearson’s χ^2^ test was applied when all expected cell frequencies were ≥5; otherwise, Fisher’s exact test was used to ensure validity. Statistical significance was set at *p* < 0.05, and odds ratios (OR) with 95% confidence intervals (CI) were calculated.

Logistic regression was not performed due to the limited sample size and wide dispersion of age, which could yield unstable estimates and convergence issues, making the model unsuitable. Normality of continuous variables was assessed using the Shapiro–Wilk test with Lilliefors correction, which confirmed non-normal distributions and further supported the use of non-parametric association tests instead of parametric regression models.

## 3. Results and Discussion

### 3.1. Characterisation of the Population

#### 3.1.1. Profile of the Studied Population

A total of 142 individuals participated in this study: 115 (81.0%) females and 27 (19.0%) males. This is consistent with a European study on sex differences, which also reported that women use psychotropic medications, including antidepressants, more frequently than men, despite similar reasons for use across sexes [21]. Conversely, research conducted in Sweden found that men report depression more frequently than women but receive fewer antidepressant prescriptions, suggesting possible under-treatment. Women, in contrast, receive more antidepressant prescriptions than men without necessarily reporting depression, which may indicate potential over-treatment [22].

In this study, participants were aged between 18 and 93 years, with a mean age of 57.8 ± 16.0 years and a median age of 58.0 years. Owing to the wide and heterogeneous distribution of age, the cohort was stratified into two groups: adults aged 18–64 years and those aged ≥65 years, a cut-off commonly applied in epidemiological and clinical research to define older adults. Among the participants, 44 (31.0%) were aged over 64 years.

Regarding antidepressant therapy, 97 patients (68.3%) were prescribed one antidepressant, whereas 45 (31.7%) received two or more. Most cases of polypragmasia involved a combination of an SSRI with trazodone (5-HT modulator).

Most patients (59.9%) had been undergoing treatment with antidepressants for at least one year but less than five years.

Table 1 presents the frequency and percentage distributions of sex, age, number of antidepressants used, and therapy duration in the studied population.

#### 3.1.2. Diagnosis

The diagnostic criteria for psychiatric disorders in this study followed the guidelines of the *Diagnostic and Statistical Manual of Mental Disorders* (DSM-5) of the American Psychiatric Association [23].

**Figure 1:**
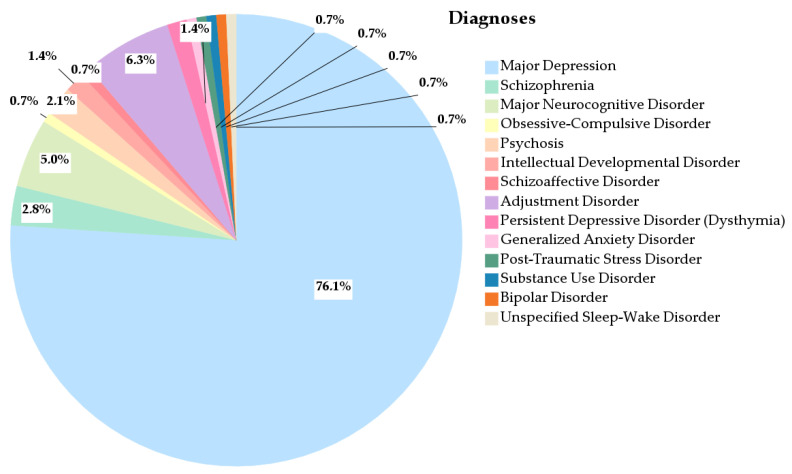
Distribution of psychiatric diagnoses in the study population.

Regarding the distribution of diagnoses among participants prescribed antidepressants, major depressive disorder was the most frequent (76.1%), followed by adjustment disorder (6.3%) and major neurocognitive disorder (5.0%) (Figure 1).

These findings are partially consistent with the study by Simon et al. [24], which reported depressive disorders as the most prevalent psychiatric diagnosis among individuals who filled antidepressant prescriptions. In their study, lower proportions of prescriptions were linked to attention deficit disorders (3%), bipolar disorders (3%), and anxiety disorders (27%). However, 39% of patients who filled antidepressant prescriptions had no documented psychiatric diagnosis [24]. In contrast, our results reveal a higher prevalence of major depressive disorder, and all individuals receiving antidepressants in our sample had a confirmed psychiatric diagnosis.

#### 3.1.3. Classes of Antidepressants

In this study, five classes of antidepressants were identified: TCAs, TeCAs and unicyclic antidepressants, SSRIs, SNRIs, and 5-HT modulators. The classification adopted followed Vanderah (2023) [25].

The most frequently prescribed antidepressants were SSRIs, representing approximately 47.4% of all prescriptions, with sertraline (20.1%) and fluoxetine (11.3%) being the most common. The second most used class was the 5-HT modulators, with trazodone alone accounting for 27.8% of prescriptions. These findings align with INFARMED data, which also report SSRIs as the most prescribed class in Portugal, exceeding 5.2 million packages dispensed in 2023. Similarly, trazodone represented over 1.6 million packages dispensed in 2023, despite being the only representative of the 5-HT modulators class [16]. By contrast, SNRIs accounted for only 6.2% of prescriptions, and TCAs for 2.6%, making them the least frequently prescribed classes in this population.

It should be noted that the frequencies reported (*n*) correspond to the total number of antidepressants prescribed (*n* = 194) across the study population (*n* = 142), as some patients received more than one antidepressant.

Table 2 presents the distribution of antidepressant prescriptions by class and individual agents.

The results obtained in our study are consistent with recent literature, which identifies SSRIs as the most frequently prescribed class for the treatment of depression when compared with TCAs, SNRIs, and other atypical antidepressants [26]. This prescribing trend may be explained by the advantages of SSRIs, which generally do not produce life-threatening adverse effects such as overdose-induced cardiotoxicity or central nervous system toxicity, since they lack receptor antagonism [26]. Furthermore, SSRIs are usually administered once daily, require less dose titration than TCAs, and are associated with better safety profiles and fewer side effects than other antidepressants. Collectively, these features make SSRIs a safer and often more effective treatment option for many patients [26].

#### 3.1.4. Other Classes of Medications

Antidepressant therapy was frequently combined with drugs from other pharmacological classes, most of which also act on the central nervous system. The most common concomitant medications were benzodiazepines (76.8%), followed by antipsychotics (48.6%), which were prescribed in nearly half of the cases.

**Figure 2:**
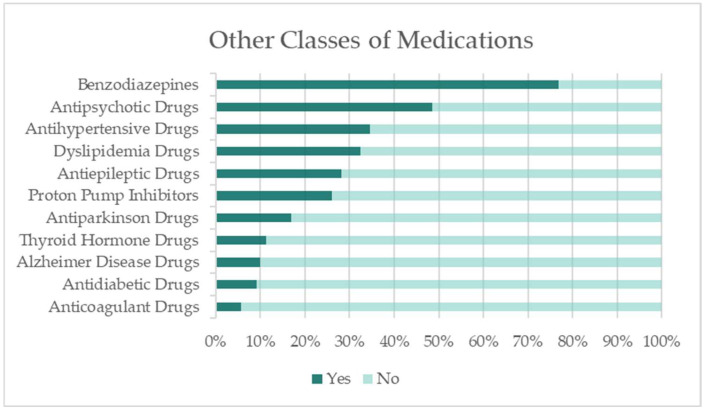
Percentage of patients using additional classes of medications.

Figure 2 presents the distribution of other classes of medications co-administered with antidepressants in the study population.

This marked combination of antidepressants with benzodiazepines was expected. At the initiation of antidepressant therapy, a benzodiazepine is sometimes added to alleviate anxiety and insomnia associated with depression, accelerate the reduction of depressive symptoms, and improve adherence to treatment [27]. However, because benzodiazepine dependence can develop rapidly, clinical guidelines recommend that their use be restricted to short-term durations [27]. In our study population, this recommendation was not followed, as the majority of patients (67.0%) reported concomitant benzodiazepine use for more than one year (χ^2^(1, n = 142) = 14.450, *p* = 0.013).

A high frequency of antipsychotic prescriptions was also observed. The literature indicates that adjunctive antipsychotics may provide a modest but significant reduction in depressive symptoms [28]. Short-term use can be appropriate in cases of treatment-resistant depression, particularly when symptoms such as intense rumination, melancholia, or severe sleep disturbances are present and show improvement with adjunctive antipsychotic therapy. However, there is no evidence to support long-term use. In our sample, a majority of patients (62.3%) reported antipsychotic use for more than one year, although this proportion was not statistically significant (χ^2^(1, n = 142) = 7.397, *p* = 0.193).

#### 3.1.5. Health and Comorbidity Profile of the Population

Among the reported health conditions, the most frequent were endocrine diseases (19.7%), emotional stress (17.6%), and cardiovascular diseases (13.4%).

The bidirectional relationship between endocrine disorders and mental illness is well established, with growing evidence supporting this interaction [29]. Mental disturbances are commonly associated with endocrine dysfunctions, while psychiatric conditions may also disrupt hormonal activity, particularly that of the thyroid and adrenal glands [29].This interplay may explain why endocrine diseases were the most frequently reported comorbidity in our population. Regarding cardiovascular conditions, depression is a recognised risk factor for poor outcomes in these patients [30]. It is estimated that around 20% of individuals with cardiovascular disease also suffer from depression [30], and our findings are consistent with these observations.

It is also important to highlight the role of emotional stress, which should be distinguished from clinical depression. Emotional stress refers to a short-term affective and physiological reaction to perceived demands or threats that typically subsides once the stressor is removed. In contrast, depression is a psychiatric disorder characterised by persistent sadness, loss of interest or pleasure, and functional impairment, lasting weeks or longer and fulfilling DSM-5 diagnostic thresholds [31]. Moreover, chronic stress—particularly when uncontrollable or socially evaluative—can dysregulate the hypothalamic–pituitary–adrenal (HPA) axis and sustain elevated pro-inflammatory cytokines (e.g., IL-1β, IL-6, TNF-α). This impairs neurogenesis, lowers neurotrophic factors such as brain-derived neurotrophic factor (BDNF), and ultimately increases vulnerability to major depressive disorder and its recurrence [32,33].

#### 3.1.6. Other Psychiatric Disorders

**Figure 3:**
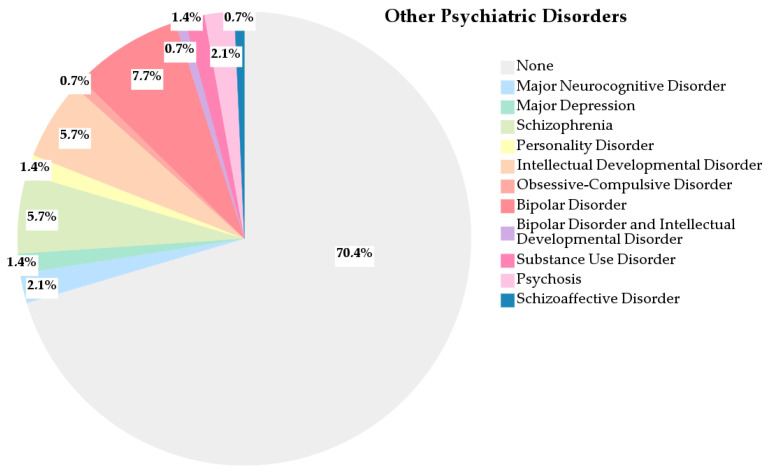
Distribution of other psychiatric disorders diagnosed in the study population.

Most individuals in our population had a single psychiatric diagnosis, with major depression being the most frequent. However, nearly one-third (29.6%) presented at least one additional psychiatric disorder. Among these, the most commonly reported were bipolar disorder (7.7%), schizophrenia (5.7%), and intellectual developmental disorder (5.7%) (Figure 3). These findings may partly explain the high prevalence of antipsychotic prescriptions observed in this population alongside antidepressant therapy.

#### 3.1.7. Adverse Effects

**Figure 4:**
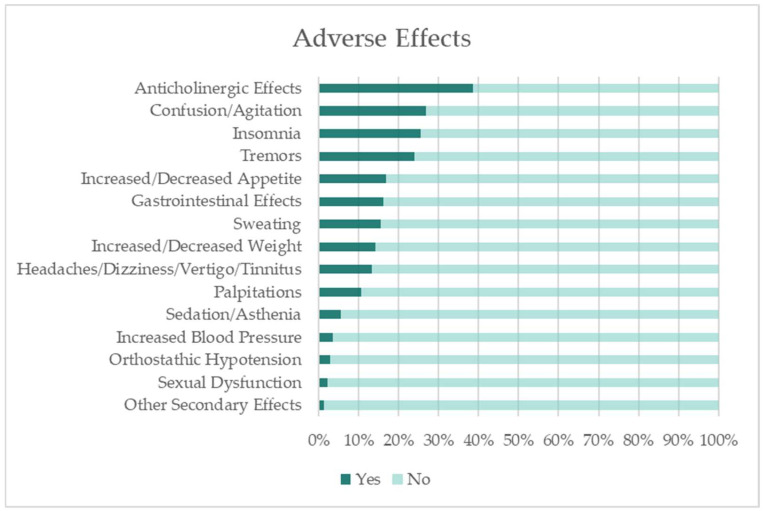
Percentage of identified adverse effects.

The most frequently reported adverse effects were anticholinergic symptoms (38.7%), confusion/agitation (26.8%), insomnia (25.4%), and tremors (23.9%) (Figure 4).

Figure 4 indicates the percentage of study participants that experienced adverse effects.

It is well established that TCAs, as well as certain antipsychotics used to manage behavioural and psychological symptoms, have anticholinergic properties [34]. Although TCAs were rarely prescribed in our sample, many patients presented polypragmasia due to comorbidities. A significant association was observed between antipsychotic use and anticholinergic effects: nearly two-thirds (63.6%) of the individuals who reported anticholinergic symptoms were medicated with antipsychotics (χ^2^(1, n = 142) = 8.134, *p* = 0.004).

Although the evaluation of benzodiazepine-related adverse effects was not a primary aim of this study, it emerged as a relevant exploratory analysis given the high prevalence of benzodiazepine use in the sample and their well-documented side effect profile. Some benzodiazepines, such as alprazolam, diazepam, flurazepam, and oxazepam, are also recognized to produce mild anticholinergic effects [34]. In our study, this association was evident: 89.1% of individuals reporting anticholinergic effects were medicated with benzodiazepines (χ^2^(1, n = 142) = 7.651, *p* = 0.006). This observation highlights the relevance of exploring adverse outcomes in the context of polypragmasia.

Insomnia was another commonly reported adverse effect. Although this can be partially explained by age—despite only 31% of the population being over 64 years old—age-related changes in sleep patterns and circadian rhythms are well documented [35]. Furthermore, several pharmacological agents, such as those with anticholinergic or antihistaminic properties, as well as anticonvulsants, antispasmodics, benzodiazepines, and opioids, may cause daytime drowsiness, thereby disrupting the normal sleep–wake cycle [35]. In our study, no significant association was observed between benzodiazepine use and reported insomnia. However, a strong association was found with antipsychotic drugs: 86.1% of the individuals who reported insomnia were under antipsychotic treatment (χ^2^(1, n = 142) = 27.177, *p* < 0.001).

Similarly, the side effect of confusion/agitation also appeared to be associated with Similarly, the side effect of confusion/agitation also appeared to be associated with antipsychotic drug use, although to a lesser extent. Among the patients reporting confusion/agitation, 65.8% were taking antipsychotics for their psychiatric disorder.

Other drug classes combined with antidepressant therapy did not demonstrate to have significant associations with the adverse effects reported by this population. Nevertheless, it is important to acknowledge that the relatively small sample size, together with the high prevalence of polypragmasia, may have influenced these observations.

### 3.2. Association Between Sex and Antidepressants Consumption

This section explores sex-related patterns in antidepressant consumption based on data collected from all patients included in this study.

Among participants taking TCAs, 100.0% were women (*p* = 0.583, Fisher’s exact test). At the sex level, this corresponded to 4.3% of women consuming TCAs and 0.0% of men. No significant differences were observed between sexes. The OR for women consuming TCAs compared to men was 1.25 (95% CI = 1.15–1.35).

Of the individuals consuming TeCAs and unicyclic antidepressants, 83.9% were women and 16.1% were men, with no statistically significant association (χ^2^(1, n = 142) = 0.214, *p* = 0.643). At the sex level, 22.6% of women and 18.5% of men used this class. The OR for women compared with men was 1.29 (95% CI = 0.44–3.73), which was not statistically significant.

For SSRIs, 76.9% of consumers were women and 23.1% were men. Although no statistically significant association was found (χ^2^(1, n = 142) = 2.716, *p* = 0.099), proportions differed: 60.9% of women and 77.8% of men used this class. The OR for men compared with women was 2.25 (95% CI = 0.84–6.00), which was not statistically significant.

Among those consuming SNRIs, 91.7% were women and 8.3% were men (*p* = 0.463, Fisher’s exact test). In terms of prevalence, 9.6% of women and 3.7% of men used SNRIs. No significant differences were observed between sexes. The OR for women compared with men was 2.75 (95% CI = 0.34–22.27), also not statistically significant.

A different pattern emerged for 5-HT modulators: 90.7% of consumers were women and 9.3% were men, with a statistically significant association (χ^2^(1, n = 142) = 5.385, *p* = 0.020). By sex, 42.6% of women and 18.5% of men consumed this class. Women were thus 3.27 times more likely to take 5-HT modulators than men (OR = 3.27, 95% CI = 1.16–9.23).

Regarding polypragmasia, among those using more than one antidepressant, 86.7% were women and 13.3% men, while for those using a single antidepressant, 78.4% were women and 21.6% men. No statistically significant association was observed (χ^2^(1, n = 142) = 1.381, *p* = 0.240). The OR for women compared with men was 1.80 (95% CI = 0.67–4.81).

Table 3 summarizes the results presented and discussed previously.

According to these results, women were consistently the predominant consumers across most antidepressant subclasses, including TCAs, TeCAs and unicyclic antidepressants, SNRIs, and 5-HT modulators. However, statistical significance was only observed for 5-HT modulators, where women were found to be 3.27 times more likely to consume trazodone than men. This finding is consistent with Estrela et al. [12], who reported that women had more than three times the prescription rates of antidepressants compared to men during the COVID-19 pandemic. Estrela et al. [4] also emphasized that women, particularly those aged 60 and above, were the primary consumers of antidepressants and anxiolytics, reinforcing the gender-related vulnerability to common mental disorders.

Interestingly, a higher proportion of men in our sample used SSRIs compared with women (77.8% vs. 60.9%, respectively). Although not statistically significant, this contrasts with the findings of Estrela et al. [4,12], who reported consistently higher use among women, without a reversal in proportional consumption. This discrepancy may reflect sample-specific dynamics or regional prescribing practices not fully captured in national datasets.

Finally, regarding the use of multiple antidepressants, 86.7% of patients using more than one were women. Although not statistically significant, this aligns with Estrela et al. [4], who highlighted polypharmacy among older adults—especially women—as a major concern due to risks of inappropriate medication and adverse effects. Our findings support these observations and underscore the importance of careful monitoring of combined antidepressant use, particularly in female populations.

### 3.3. Association Between Age and Antidepressants Consumption

This section explores the relationship between antidepressant consumption and the age of participants in this study.

For TCAs, 60.0% of consumers were over 64 years old and 40.0% were aged between 18 and 64 years (*p* = 0.173, Fisher’s exact test). By age group, 6.8% of patients older than 64 consumed TCAs, compared with 2.0% of those aged 18–64. No statistically significant differences were observed. The OR for patients >64 compared with those aged 18–64 was 3.51 (95% CI = 0.57–21.81), which was not statistically significant.

Among individuals consuming TeCAs and unicyclic antidepressants, 51.6% were older than 64 and 48.4% were aged 18–64. Within age groups, 36.4% of patients >64 years used this class compared with 15.3% of patients aged 18–64. A statistically significant association was observed (χ^2^(1, n = 142) = 7.890, *p* = 0.005). Patients older than 64 were **3.16 times more likely** to consume TeCAs and unicyclic antidepressants than younger patients (OR = 3.16, 95% CI = 1.39–7.21).

For SSRIs, 73.6% of consumers were aged 18–64 and 26.4% were >64 years. By group, 68.4% of patients aged 18–64 and 54.4% of those >64 consumed SSRIs. No significant association was found (χ^2^(1, n = 142) = 2.521, *p* = 0.112). The OR for younger versus older patients was 1.80 (95% CI = 0.87–3.74), not statistically significant.

In the case of SNRIs, 75.0% of consumers were aged 18–64 and 25.0% were >64 (*p* = 0.754, Fisher’s exact test). By group, 9.2% of patients aged 18–64 and 6.8% of patients >64 used SNRIs. No significant differences were observed. The OR for younger versus older patients was 1.38 (95% CI = 0.36–5.37), also not significant.

For 5-HT modulators, 42.6% of consumers were older than 64 and 57.4% were aged 18–64. By group, 52.3% of patients >64 and 31.6% of those aged 18–64 used this class. A statistically significant association was observed (χ^2^(1, n = 142) = 5.489, *p* = 0.019). Patients over 64 were **2.37 times more likely** to consume 5-HT modulators than younger patients (OR = 2.37, 95% CI = 1.14–4.91).

Regarding polypragmasia, 48.9% of patients using more than one antidepressant were >64 and 51.1% were aged 18–64. Within groups, 50.0% of older patients used multiple antidepressants, compared with 23.5% of younger patients. A statistically significant association was observed (χ^2^(1, n = 142) = 9.874, *p* = 0.002). Patients >64 were **3.26 times more likely** to use more than one antidepressant than those aged 18–64 (OR = 3.26, 95% CI = 1.54–6.93).

Table 4 provides a summary of the results previously presented and discussed.

The present study shows that patients aged over 64 years were significantly more likely to consume certain classes of antidepressants, particularly TeCAs and unicyclic antidepressants, and 5-HT modulators, compared with those aged 18–64. Additionally, older patients were significantly more likely to be prescribed multiple antidepressants simultaneously, with an OR of 3.26. These findings are consistent with Negrão et al. [14], who reported a positive correlation between the percentage of elderly residents in a municipality and the increase in antidepressant consumption following the COVID-19 pandemic, reinforcing the age-related trend.

Estrela et al. [4] also supports these conclusions, reporting that more than half of antidepressant consumers in Portugal are aged 60 years or older. Their study highlighted concerns about excessive use and polypragmasia in older populations, particularly regarding benzodiazepines and antidepressants. It also noted that TCAs are less frequently prescribed due to their adverse effects, consistent with our observation of their limited use and lack of statistical significance.

Finally, Madeira et al. [5] confirmed that individuals over 50 years old account for more than half of antidepressant prescriptions in Portugal, documenting a 47% increase in consumption between 2016 and 2019. SSRIs were the most prescribed class, while TCAs declined, and drugs such as trazodone and mirtazapine—commonly used in older patients, often for off-label indications like insomnia—were increasingly preferred. That study also emphasised the prevalence of polypragmasia and the central role of general practitioners in prescribing psychotropic medications, which may contribute to the patterns observed in our sample.

### 3.4. Association Between Antidepressants Consumption and Adverse Effects

This section explores in further detail the association between antidepressant consumption and reported adverse effects, based on the data collected from all study participants. The analysis was stratified by antidepressant class.

Among patients using TeCAs and unicyclic antidepressants, 54.8% reported anticholinergic effects, while 45.2% did not. Of those who experienced such effects, 30.9% were users of this class, compared with 16.1% of patients who did not report them. A statistically significant association was confirmed (χ^2^(1, n = 142) = 4.335, *p* = 0.037). Odds ratio analysis indicated that patients were 2.33 times more likely to experience anticholinergic effects when taking these drugs (OR = 2.33, 95% CI = 1.04–5.28).

Similarly, tremors were reported by 38.7% of TeCA/unicyclic users. At the population level, 35.3% of individuals with tremors consumed this class, compared with 17.6% of those without. Again, a significant association was observed (χ^2^(1, n = 142) = 4.748, *p* = 0.029), with an OR of 2.56 (95% CI = 1.08–6.04).

Gastrointestinal effects were mentioned by 32.3% of patients using these antidepressants. At the population level, 43.5% of individuals reporting gastrointestinal complaints were TeCA/unicyclic users. This association was statistically significant (χ^2^(1, n = 142) = 7.536, *p* = 0.006), with an OR of 3.59 (95% CI = 1.39–9.28).

Orthostatic hypotension was less frequent (9.7% of users), yet 75.0% of patients who reported this effect were taking TeCAs/unicyclic antidepressants. Although the sample size was small, Fisher’s exact test revealed a significant association (*p* = 0.033), with an OR of 11.79 (95% CI = 1.18–117.66).

These findings are consistent with the literature. Bupropion is associated with dry mouth, nausea, tremors, dizziness and gastrointestinal disturbances [36]; maprotiline with drowsiness, tremors, gastrointestinal effects and orthostatic hypotension [37]; and mirtazapine with drowsiness, tremors, nausea/vomiting and confusion [38].

Among SSRI users, 27.5% reported anticholinergic effects, while 72.5% did not. At the population level, 45.5% of patients reporting anticholinergic symptoms were taking SSRIs. A significant association was confirmed (χ^2^(1, n = 142) = 13.537, *p* < 0.001). OR analysis showed that patients not taking SSRIs were 3.77 times more likely to experience these effects (OR = 3.77, 95% CI = 1.83–7.77).

Other adverse effects showed a similar pattern, with lower reporting among SSRI users than non-users: confusion/agitation: OR = 2.62 (95% CI = 1.22–5.61), χ^2^ = 6.299, *p* = 0.012; insomnia: OR = 3.02 (95% CI = 1.39–6.59), χ^2^ = 8.082, *p* = 0.004; tremors: OR = 3.55 (95% CI = 1.59–7.90), χ^2^ = 10.193, *p* = 0.001; appetite changes: OR = 3.80 (95% CI = 1.52–9.48), χ^2^ = 8.868, *p* = 0.003; gastrointestinal effects: OR = 3.45 (95% CI = 1.37–8.68), χ^2^ = 7.425, *p* = 0.006; weight changes: OR = 3.19 (95% CI = 1.21–8.44), χ^2^ = 5.867, *p* = 0.015. In all cases, non-users were more likely to report these adverse effects than SSRI users. This aligns with the safer profile attributed to SSRIs compared to older antidepressant classes, despite the fact that citalopram, fluoxetine, fluvoxamine, paroxetine and sertraline are all documented to potentially cause these effects [39,40,41,42,43].

However, participants in this study who consumed SSRIs reported fewer of these adverse effects than those who did not consume them, which led to statistically significant results, but with OR values favouring patients taking SSRIs over those taking other classes of antidepressants. These results are in line with the wide prescription of SSRIs for depression, since this class is associated with better safety. Although SSRIs can also cause undesirable effects, these are considered new generation antidepressants, which makes them safer and able to cause fewer adverse effects compared to the other classes of antidepressants [26].

For 5-HT modulators (primarily trazodone), a higher burden of adverse effects was observed. Among users, 64.8% reported anticholinergic effects. Of all patients with such complaints, 63.6% were trazodone users, corresponding to a strong association (χ^2^(1, n = 142) = 24.980, *p* < 0.001) with an OR of 6.26 (95% CI = 2.96–13.24).

Other significant associations for trazodone included: confusion/agitation: OR = 3.61 (95% CI = 1.67–7.83), χ^2^ = 11.144, *p* = 0.001; insomnia: OR = 3.10 (95% CI = 1.42–6.75), χ^2^ = 8.437, *p* = 0.004; tremors: OR = 3.67 (95% CI = 1.64–8.20), χ^2^ = 10.688, *p* = 0.001; appetite changes: OR = 4.21 (95% CI = 1.66–10.70), χ^2^ = 10.051, *p* = 0.002; gastrointestinal effects: OR = 2.47 (95% CI = 1.00–6.13), χ^2^ = 3.983, *p* = 0.046; sweating: OR = 3.50 (95% CI = 1.36–9.03), χ^2^ = 7.244, *p* = 0.007; weight changes: OR = 4.78 (95% CI = 1.71–13.38), χ^2^ = 10.097, *p* = 0.001; headaches/dizziness/vertigo/tinnitus: OR = 4.33 (95% CI = 1.54–12.23), χ^2^ = 8.598, *p* = 0.003 and palpitations: OR = 5.37 (95% CI = 1.62–17.87), χ^2^ = 8.871, *p* = 0.003.

These results are in agreement with trazodone’s known profile, which includes frequent undesirable effects such as confusion, insomnia, dizziness, tremors, palpitations, gastrointestinal symptoms and hyperhidrosis [44].

For TCAs and SNRIs, no statistically significant results were observed in relation to the considered adverse effects. This may be explained by their low prevalence of use in the studied population, limiting statistical power.

Overall, significant associations were identified mainly for TeCAs/unicyclic antidepressants and 5-HT modulators, where several adverse effects were more frequent among users. In contrast, SSRIs showed an inverse pattern, with most adverse effects more frequently reported by non-users, reinforcing their comparatively safer tolerability profile.

Table 5 presents a complete overview of the statistical associations between antidepressant use and adverse effects.

## 4. Study Limitations, Public Health Implications, and Future Perspectives

This study presents several limitations that must be acknowledged when interpreting its findings. The sample size, although adequate for exploratory analysis, may not provide sufficient statistical power to detect subtle associations or allow for subgroup analyses. While the study focused on a population from an inland region of Portugal, yielding geographically and demographically more restricted findings, this may limit the generalisability of the results to broader or more diverse populations. Reliance on data reported by healthcare professionals with detailed knowledge of patients’ clinical histories substantially reduces, though does not entirely eliminate, the potential for recall bias, particularly concerning the timing and extent of exposure and symptom onset in outpatient cases. Furthermore, the cross-sectional design precludes the establishment of temporal or causal relationships, thereby requiring cautious interpretation of the observed associations.

Despite these limitations, the results carry significant public health implications. The observed patterns highlight a potentially under-recognised burden associated with medication use in patients with the underlying disease. This is particularly concerning in vulnerable populations, where the cumulative effects of polypragmasia, adverse drug reactions, and interactions may be compounded by limited access to regular medical follow-up and medication review. The evidence underscores the urgent need for targeted interventions, including the optimisation of prescribing practices, strengthened pharmacovigilance systems, and improved patient education to promote safe and effective medication use. Moreover, the findings can support healthcare planning and inform clinical guidelines, ensuring that therapeutic strategies are adapted to the specific needs and risk profiles of these patient groups.

In light of the evidence, several measures should be prioritised. Regular monitoring of medication use and patient responses in clinical settings would facilitate the early identification of adverse drug reactions and potential medication-related complications. Patient-focused educational initiatives could enhance adherence, awareness of potential side effects, and safe medication management strategies. Healthcare systems should strengthen pharmacovigilance practices and ensure that clinical guidelines are informed by local patient profiles and medication usage patterns. In addition, healthcare professionals should receive continuous training and resources to recognise early signs of medication-related problems, enabling timely and effective intervention. These measures are actionable, context-specific, and supported by the present findings.

Future research should address the limitations of the current study by employing longitudinal designs to establish causal relationships and temporal patterns between medication use and disease progression. Larger, multicentre studies including diverse patient populations across different demographic and geographic contexts will enhance the generalisability of the results. Incorporating objective biomarkers and advanced analytical methods would enable a more precise assessment of medication exposure and its biological effects. Furthermore, involving patients and healthcare providers through participatory research approaches could improve data accuracy, foster trust, and facilitate the translation of scientific evidence into clinical practice and health policy, ultimately optimising medication safety and efficacy.

## 5. Conclusions

With the progressive increase in the prescription and consumption of antidepressants in recent years, this study sought to characterise the profile of the Beira Interior population regarding the use of this therapeutic class. In addition to mapping the overall consumption trends, it was possible to draw conclusions about associations with sex, age, and the adverse effects reported.

The findings reveal a marked predominance of women (81.0%) in antidepressant use, consistent with international patterns, though the possibility of underdiagnosis and undertreatment in men, alongside potential overtreatment in women, warrants further investigation. Major depressive disorder was the leading diagnosis (76.1%) underlying antidepressant prescriptions, at higher rates than those reported in comparable studies. SSRIs and serotonin modulators, particularly trazodone, predominated in prescribing patterns, while TCAs and SNRIs were less frequently used. A notable finding was the extensive and prolonged co-administration of benzodiazepines (76.8%), with a significant proportion of patients exceeding one year of use—contrary to clinical guidelines and increasing the risk of dependence and adverse outcomes. Additionally, nearly half of the population (48.6%) received concomitant antipsychotics, often for extended periods, despite limited evidence supporting this practice and the elevated risk of serious side effects. High incidences of anticholinergic and neurological adverse effects were linked to polypragmasia, highlighting the toxicity burden.

In relation to sex, women were significantly more likely to consume serotonin modulators compared with men (OR = 3.27, *p* = 0.020). For other antidepressant classes, sex differences were not statistically significant, suggesting that sex has a limited and variable influence on antidepressant consumption. This underlines the need to further explore other clinical and pharmacokinetic factors that may contribute to differential treatment responses. With regard to age, individuals over 64 years were significantly more likely to consume tetracyclic/unicyclic antidepressants (OR = 3.16, *p* = 0.005), serotonin modulators (OR = 2.37, *p* = 0.019), and multiple antidepressants (OR = 3.26, *p* = 0.002) compared to younger adults. These results emphasise the clinical complexity of older patients and the need for age-tailored prescribing and closer monitoring of adverse outcomes.

The data further indicate significant associations between the use of tetracyclic/unicyclic antidepressants and serotonin modulators and the occurrence of multiple adverse effects, including anticholinergic effects, tremors, gastrointestinal symptoms, confusion, insomnia, appetite changes, and orthostatic hypotension, with odds ratios ranging from 2.33 to 11.79. Conversely, SSRIs were associated with a lower incidence of adverse effects compared to other classes, reflecting their favourable safety profile. TCAs and SNRIs showed no significant associations, likely reflecting their lower use in the studied sample. These results corroborate the known safety and risk profiles of antidepressants and highlight the need for rigorous monitoring of adverse events in polypharmacy, with particular attention to vulnerable populations.

The findings have direct implications for clinical practice. Healthcare providers should prioritise individualised prescribing strategies that account for patient sex, age, comorbidities, and polypragmasia risks. In particular, older patients require enhanced monitoring to detect early signs of adverse effects and adjust therapy proactively. Regular medication reviews, combined with structured adverse event reporting systems, may reduce preventable harms and improve adherence. Furthermore, the high prevalence of polypragmasia and multiple antidepressant use underscores the importance of deprescribing protocols and therapeutic drug monitoring within mental health care. From a public health and policy perspective, the results highlight the need for regional prescription monitoring programmes capable of detecting prescribing trends and potential overuse. Policymakers should implement targeted educational initiatives for prescribers to reinforce evidence-based antidepressant use, particularly in high-risk populations. The integration of pharmacists into multidisciplinary mental health teams could further strengthen medication safety and optimise therapeutic outcomes.

This study also presents limitations, including the modest sample size, the wide age span within the 18–64 years category that limited subgroup analyses, and the low representativeness of some antidepressant classes. Future research should build on these findings by recruiting larger and more representative samples, enabling more refined age stratification and analysis of less frequently prescribed antidepressants. Longitudinal designs will be essential to establish causal relationships between antidepressant use and adverse outcomes and to evaluate the effectiveness of interventions aimed at optimising pharmacotherapy while minimising harm.

Nevertheless, the present research establishes an important regional benchmark for antidepressant consumption, providing evidence that can guide clinicians, inform health policies, and shape priorities for future investigation in psychopharmacology and mental health care. Importantly, the study underscores the concomitant use of multiple antidepressants and other psychotropic drugs, as well as the impact of adverse effects on patients’ quality of life, reinforcing the need for systematic monitoring and careful management of antidepressant therapy in clinical practice.

## Figures and Tables

**Table 1:** Demographic and treatment characteristics of the study population: sex, age, number of antidepressants prescribed, and duration of therapy.

Variable	*n*	%
**Sex**		
Female	115	81.0
Male	27	19.0
**Age (in years)**		
18–64	98	69.0
>64	44	31.0
**Number of antidepressants**		
1	97	68.3
>1	45	31.7
**Therapy duration**		
<1 month	9	6.3
<6 months	19	13.4
<1 year	12	8.5
≥1 year	85	59.9
≥5 years	14	9.9
Not specified	3	2.1

**Table 2:** Antidepressants (in their classes) consumption distribution.

Classes	Antidepressants	Count *n* (%)	Classes of Antidepressants *n* (%)
**TCAs**	Amitriptyline	3 (1.5)	5 (2.6)
	Clomipramine	2 (1.0)	
**TeCAs and unicyclic**	Bupropion	5 (2.6)	31 (16.0)
	Maprotiline	1 (0.5)	
	Mirtazapine	25 (12.9)	
**SSRIs**	Citalopram	12 (6.2)	92 (47.4)
	Fluoxetine	22 (11.3)	
	Fluvoxamine	3 (1.5)	
	Paroxetine	16 (8.2)	
	Sertraline	39 (20.1)	
**SNRIs**	Duloxetine	4 (2.1)	12 (6.2)
	Venlafaxine	8 (4.1)	
**5-HT modulators**	Trazodone	54 (27.8)	54 (27.8)

**Table 3:** Association between sex and antidepressant consumption in the study population.

Variable	*n* (Yes)	*n* (No)	*p*-Value	OR (95% CI)
**TCAs**				
Female Male Total	505	110 27 137	0.583	1.25 (1.15–1.35) ^a^
**TeCAs and unicyclic**				
Female Male Total	26 5 31	89 22 111	0.643	1.29 (0.44–3.73) ^a^
**SSRIs**				
Female Male Total	70 21 91	45 6 51	0.099	2.25 (0.84–6.00) ^b^
**SNRIs**				
Female Male Total	11 1 12	104 26 130	0.463	2.75 (0.34–22.27) ^a^
**5-HT modulators**				
Female Male Total	49 5 54	66 22 88	**0.020**	3.27 (1.16–9.23) ^a^
**Variable**	***n*** **(>1)**	***n*** **(1)**	***p*** **-Value**	**OR (95% CI)**
**Number of antidepressants**				
Female Male Total	39 6 45	76 21 97	0.240	1.80 (0.67–4.81) ^c^

^a^ OR Female/Male; ^b^ OR Male/Female; ^c^ OR > 1 antidepressant/1 antidepressant.

**Table 4:** Association between age and antidepressant consumption in the study population.

Variable	*n* (Yes)	*n* (No)	*p*-Value	OR (95% CI)
**TCAs**				
18–64 >64 Total	2 3 5	96 41 137	0.173	3.51 (0.57–21.81) ^a^
**TeCAs and unicyclic**				
18–64 >64 Total	15 16 31	83 28 111	**0.005**	3.16 (1.39–7.21) ^a^
**SSRIs**				
18–64 >64 Total	67 24 91	31 20 51	0.112	1.80 (0.87–3.74) ^b^
**SNRIs**				
18–64 >64 Total	9 3 12	89 41 130	0.754	1.38 (0.36–5.37) ^b^
**5-HT modulators**				
18–64 >64 Total	31 23 54	67 21 88	**0.019**	2.37 (1.14–4.91) ^a^
**Variable**	***n*** **(>1)**	***n*** **(1)**	***p*** **-Value**	**OR (95% CI)**
**Number of antidepressants**				
18–64 >64 Total	23 22 45	75 22 97	**0.002**	3.26 (1.54–6.93) ^c^

^a^ OR > 64/18–64; ^b^ 18–64/> 64; ^c^ OR > 1 antidepressant/1 antidepressant.

**Table 5:** Association results for antidepressant consumption according to adverse effects in a population undergoing treatment with this class of medication.

Variable 1	Variable 2	*n* (Yes)	*n* (No)	*p*-Value	OR (95% CI)
**TeCAs and** **unicyclic**	**Anticholinergic effects**				
	Yes No Total	17 14 31	38 73 111	**0.037**	2.33 (1.04–5.28) ^a^
	**Tremors**				
	Yes No Total	12 19 31	22 89 111	**0.029**	2.56 (1.08–6.04) ^a^
	**Gastrointestinal effects**				
	Yes No Total	10 21 31	13 98 111	**0.006**	3.59 (1.39–9.28) ^a^
	**Orthostatic hypotension**				
	Yes No Total	3 28 31	1 110 111	**0.033**	11.79 (1.18–117.66) ^a^
**SSRIs**	**Anticholinergic effects**				
	Yes No Total	25 66 91	30 21 51	**<0.001**	3.77 (1.83–7.77) ^b^
	**Confusion/Agitation**				
	Yes No Total	18 73 91	20 31 51	**0.012**	2.62 (1.22–5.61) ^b^
	**Insomnia**				
	Yes No Total	16 75 91	20 31 51	**0.004**	3.02 (1.39–6.59) ^b^
	**Tremors**				
	Yes No Total	14 77 91	20 31 51	**0.001**	3.55 (1.59–7.90) ^b^
	**Increased/Decreased appetite**				
	Yes No Total	9 82 91	15 36 51	**0.003**	3.80 (1.52–9.48) ^b^
	**Gastrointestinal effects**				
	Yes No Total	9 82 91	14 37 51	**0.006**	3.45 (1.37–8.68) ^b^
	**Increased/Decreased weight**				
	Yes No Total	8 83 91	12 39 51	**0.015**	3.19 (1.21–8.44) ^b^
**5-HT modulators**	**Anticholinergic effects**				
	Yes No Total	35 19 54	20 68 88	**<0.001**	6.26 (2.96–13.24) ^a^
	**Confusion/Agitation**				
	Yes No Total	23 31 54	15 73 88	**0.001**	3.61 (1.67–7.83) ^a^
	**Insomnia**				
	Yes No Total	21 33 54	15 73 88	**0.004**	3.10 (1.42–6.75) ^a^
	**Tremors**				
	Yes No Total	21 33 54	13 75 88	**0.001**	3.67 (1.64–8.20) ^a^
	**Increased/Decreased appetite**				
	Yes No Total	16 38 54	8 80 88	**0.002**	4.21 (1.66–10.70) ^a^
	**Gastrointestinal effects**				
	Yes No Total	13 41 54	10 78 88	**0.046**	2.47 (1.00–6.13) ^a^
	**Sweating**				
	Yes No Total	14 40 54	8 80 88	**0.007**	3.50 (1.36–9.03) ^a^
	**Increased/Decreased weight**				
	Yes No Total	14 40 54	6 82 88	**0.001**	4.78 (1.71–13.38) ^a^
	**Headaches/Dizziness/Vertigo/Tinnitus**				
	Yes No Total	13 41 54	6 82 88	**0.003**	4.33 (1.54–12.23) ^a^
	**Palpitations**				
	Yes No Total	11 43 54	4 84 88	**0.003**	5.37 (1.62–17.87) ^a^

^a^ OR Yes/No; ^b^ OR No/Yes.

## Data Availability

The original contributions presented in this study are included in the article/Supplementary Material. Further inquiries can be directed to the corresponding author(s).

## Supplementary Materials

The following supporting information can be downloaded at: https://www.mdpi.com/article/10.3390/healthcare13172177/s1, Survey Sample S1: Questionário de Adesão.

## Abbreviations

The following abbreviations are used in this manuscript:

5-HT modulators	Serotonin Modulators
INFARMED	Autoridade Nacional do Medicamento e Produtos de Saúde, I.P.
OECD	Organisation for Economic Cooperation and Development
OR	Odds Ratio
SSRIs	Selective Serotonin Reuptake Inhibitors
SNRIs	Serotonin and Norepinephrine Reuptake Inhibitors
TeCAs	Tetracyclic Antidepressants
TCAs	Tricyclic Antidepressants

## References

[1] Malhi G.S., Mann J.J. (2018). Depression. Lancet.

[2] Bessa J.M., Carvalho S., Cunha I.B., Fernandes M., Matos-Pires A., Neves R., Oliveira-Maia A.J., Santos S., Santos V. (2022). Treatment-Resistant Depression in Portugal: Perspective From Psychiatry Experts. Front. Psychiatry.

[3] Antunes A., Frasquilho D., Azeredo-Lopes S., Neto D., Silva M., Cardoso G., Caldas-de-Almeida J.M. (2018). Disability and common mental disorders: Results from the World Mental Health Survey Initiative Portugal. Eur. Psychiatry.

[4] Estrela M., Herdeiro M.T., Ferreira P.L., Roque F. (2020). The Use of Antidepressants, Anxiolytics, Sedatives and Hypnotics in Europe: Focusing on Mental Health Care in Portugal and Prescribing in Older Patients. Int. J. Environ. Res. Public Health.

[5] Madeira L., Queiroz G., Henriques R. (2022). Psychotropic drugs in Portugal from 2016 to 2019: A nationwide pharmacoepidemiological profile. medRxiv.

[6] World Health Organization (2018). Management of Physical Health Conditions in Adults with Severe Mental Disorders: WHO Guidelines.

[7] World Health Organization (2021). Comprehensive Mental Health Action Plan 2013–2030.

[8] Alabaku O., Yang A., Tharmarajah S., Suda K., Vigod S., Tadrous M. (2023). Global trends in antidepressant, atypical antipsychotic, and benzodiazepine use: A cross-sectional analysis of 64 countries. PLoS ONE.

[9] Lunghi C., Dugas M., Leclerc J., Poluzzi E., Martineau C., Carnovale V., Stéfan T., Blouin P., Lépine J., Jalbert L. (2022). Global prevalence of antidepressant drug utilization in the community: Protocol for a systematic review. BMJ Open.

[10] Gutiérrez-Abejón E., Herrera-Gómez F., Criado-Espegel P., Álvarez F.J. (2020). Trends in Antidepressants Use in Spain between 2015 and 2018: Analyses from a Population-Based Registry Study with Reference to Driving. Pharmaceuticals.

[11] World Health Organization (2022). COVID-19 Pandemic Triggers 25% Increase in Prevalence of Anxiety and Depression Worldwide.

[12] Estrela M., Silva T.M., Gomes E.R., Piñeiro M., Figueiras A., Roque F., Herdeiro M.T. (2022). Prescription of anxiolytics, sedatives, hypnotics and antidepressants in outpatient, universal care during the COVID-19 pandemic in Portugal: A nationwide, interrupted time-series approach. J. Epidemiol. Community Health.

[13] Yao H., Chen J.H., Xu Y.F. (2020). Patients with mental health disorders in the COVID-19 epidemic. Lancet Psychiatry.

[14] Negrão L.G., Coelho C., Castel-Branco M.M., Figueiredo I.V., Fernandez-Llimos F. (2024). Impact of the COVID-19 pandemic on antidepressant consumption in the Central region of Portugal: Interrupted time series. Soc. Psychiatry Psychiatr. Epidemiol..

[15] Madeira L., Queiroz G., Henriques R. (2023). Prepandemic psychotropic drug status in Portugal: A nationwide pharmacoepidemiological profile. Sci. Rep..

[16] (2024). Autorização de Introdução No Mercado—INFARMED, I.P. https://www.infarmed.pt/web/infarmed/entidades/medicamentos-uso-humano/autorizacao-de-introducao-no-mercado.

[17] Afonso R.M., Ribeiro O., Vaz Patto M., Loureiro M., Loureiro M.J., Castelo-Branco M., Patrício S., Alvarinhas S., Tomáz T., Rocha C. (2018). Reaching 100 in the Countryside: Health Profile and Living Circumstances of Portuguese Centenarians from the Beira Interior Region. Curr. Gerontol. Geriatr. Res..

[18] Naslund J.A., Deng D. (2021). Addressing mental health stigma in low-income and middle-income countries: A new frontier for digital mental health. Ethics Med. Public Health.

[19] Gomes D., Placido A.I., Mó R., Simões J.L., Amaral O., Fernandes I., Lima F., Morgado M., Figueiras A., Herdeiro M.T. (2019). Daily Medication Management and Adherence in the Polymedicated Elderly: A Cross-Sectional Study in Portugal. Int. J. Environ. Res. Public Health.

[20] de Simoes J.A., Augusto G.F., Fronteira I., Hernandez-Quevedo C. (2017). Portugal: Health System Review. Health Syst. Transit..

[21] Boyd A., Van de Velde S., Pivette M., Have M.T., Florescu S., O’Neill S., Caldas-de-Almeida J.M., Vilagut G., Haro J.M., Alonso J. (2015). Gender differences in psychotropic use across Europe: Results from a large cross-sectional, population-based study. Eur. Psychiatry.

[22] Sundbom L.T., Bingefors K., Hedborg K., Isacson D. (2017). Are men under-treated and women over-treated with antidepressants? Findings from a cross-sectional survey in Sweden. BJPsych Bull..

[23] American Psychiatric Association (2013). Diagnostic and Statistical Manual of Mental Disorders.

[24] Simon G.E., Stewart C., Beck A., Ahmedani B.K., Coleman K.J., Whitebird R.R., Lynch F., Owen-Smith A.A., Waitzfelder B.E., Soumerai S.B. (2014). National prevalence of receipt of antidepressant prescriptions by persons without a psychiatric diagnosis. Psychiatr. Serv..

[25] Vanderah T.W., Vanderah T.W. (2023). Drugs that Act in the Central Nervous System: Antidepressant Agents. Katzung’s Basic & Clinical Pharmacology.

[26] Wang S.-M., Han C., Bahk W.-M., Lee S.-J., Patkar A.A., Masand P.S., Pae C.-U. (2018). Addressing the Side Effects of Contemporary Antidepressant Drugs: A Comprehensive Review. Chonnam Med. J..

[27] Bushnell G.A., Stürmer T., Gaynes B.N., Pate V., Miller M. (2017). Simultaneous Antidepressant and Benzodiazepine New Use and Subsequent Long-term Benzodiazepine Use in Adults With Depression, United States, 2001–2014. JAMA Psychiatry.

[28] Mulder R., Hamilton A., Irwin L., Boyce P., Morris G., Porter R.J., Malhi G.S. (2018). Treating depression with adjunctive antipsychotics. Bipolar Disord..

[29] Salvador J., Gutierrez G., Llavero M., Gargallo J., Escalada J., López J., Portincasa P., Frühbeck G., Nathoe H.M. (2021). Endocrine Disorders and Psychiatric Manifestations. Endocrinology and Systemic Diseases.

[30] Li X., Zhou J., Wang M., Yang C., Sun G. (2023). Cardiovascular disease and depression: A narrative review. Front. Cardiovasc. Med..

[31] Akil H., Nestler E.J. (2023). The neurobiology of stress: Vulnerability, resilience, and major depression. Proc. Natl. Acad. Sci. USA.

[32] Hassamal S. (2023). Chronic stress, neuroinflammation, and depression: An overview of pathophysiological mechanisms and emerging anti-inflammatories. Front. Psychiatry.

[33] Sălcudean A., Popovici R.A., Pitic D.E., Sârbu D., Boroghina A., Jomaa M., Salehi M.A., Kher A.A.M., Lica M.M., Bodo C.R. (2025). Unraveling the Complex Interplay Between Neuroinflammation and Depression: A Comprehensive Review. Int. J. Mol. Sci..

[34] Tune L.E. (2001). Anticholinergic effects of medication in elderly patients. J. Clin. Psychiatry.

[35] Cohen Z.L., Eigenberger P.M., Sharkey K.M., Conroy M.L., Wilkins K.M. (2022). Insomnia and Other Sleep Disorders in Older Adults. Psychiatr. Clin. N. Am..

[36] Food and Drug Administration (2017). Highlights of Prescribing Information: Wellbutrin (Bupropion Hydrochloride).

[37] Food and Drug Administration (2014). Prescribing Information: Maprotiline Hydrochloride.

[38] Food and Drug Administration (2020). Highlights of Prescribing Information: Remeron (Mirtazapine).

[39] Food and Drug Administration (2023). Highlights of Prescribing Information: Citalopram.

[40] Food and Drug Administration (2017). Highlights of Prescribing Information: Prozac (Fluoxetine Hydrochloride).

[41] Food and Drug Administration (2017). Highlights of Prescribing Information: Luvox CR (Fluvoxamine Maleate).

[42] Food and Drug Administration (2017). Highlights of Prescribing Information: Paroxetine.

[43] Food and Drug Administration (2016). Highlights of Prescribing Information: Zoloft (Sertraline Hydrochloride).

[44] Food and Drug Administration (2017). Highlights of Prescribing Information: Desyrel (Trazodone Hydrochloride).

