# Supplementary material for: Characterization of Antidepressant Consumption in a Portuguese Inland Population
Source: Healthcare (Basel). 2025 Aug 31;13(17):2177. doi: 10.3390/healthcare13172177 (PMC12428140; doi:10.3390/healthcare13172177)
Supplement: Supplementary file 1 [file healthcare-13-02177-s001.zip › healthcare-3780855-supplementary.pdf]

Healthcare

Supplementary Material

Characterization of Antidepressant Consumption in a Portuguese Inland Population

Sofia Soares, Tiago Rosado, Vítor Hugo Santos, Cristina Rei, Patricia Amantegui, António Pissarra da Costa, Telma Chaves, Rita Valente, Fábio Duarte, Susana Pacheco, Marco Martins, Kátia Dias, Patricia Costa, Rui Costa, Sílvia Castro, Diana Sousa, Diana Figueiredo, Isabel Soares, Salomé Mouta, Bianca Jesus, Ana Pires, Cândida Ribeiro, Sónia Lobo, Leonor Correia, Sofia Malés, Fátima Vale, Carina Moita, Carolina Moura, Joana Sousa, Luís Rafael Afonso, Rita Santinho Costa, Mário Barroso and Eugenia Gallardo

FACULDADE CIÊNCIAS DA SAÚDE
Departamento de Ciências Médicas

A AMOSTRA DE FLUIDO ORAL COMO FERRAMENTA PARA A MONITORIZAÇÃO DE ANTIDEPRESSIVOS NO ÂMBITO CLÍNICO E FORENSE

Nº DE IDENTIFICAÇÃO DA AMOSTRA:

____

QUESTIONÁRIO DE ADESÃO

INQUÉRITO PARA RECOLHA DE DADOS

Este questionário, de carácter anónimo e confidencial, realizado pelo Centro de Investigação em Ciências da Saúde da Universidade da Beira Interior, visa recolher informações sobre o tratamento com fármacos antidepressivos em doentes medicados com os mesmos. Os dados recolhidos serão tratados estatisticamente com a finalidade de elaborar uma tese de doutoramento, pelo que as informações obtidas não serão facultadas a outras entidades, que escapem ao âmbito desta investigação.

Idade: ____________________ ***Sexo:*** ☐ Feminino ☐ Masculino

Hora da recolha da amostra: ____________________ ***Data:*** ____________________

Doença para a qual toma esta medicação: ____

Medicação a ser administrada para esta doença *(nome de princípio ativo ou marca comercial)* ***indicando a dose administrada, pauta e quando tomou o medicamento pela última vez (dia e hora). Indicar data de início do tratamento:***

____
____
____
____
____
____
____
____

Outra medicação a ser administrada: *(nome de princípio ativo ou marca comercial).* ***Indicar dose administrada, pauta e quando tomou o medicamento pela última vez. Indicar data de início do tratamento:***

____
____
____
____
____
____
____
____
____
____

SAMPLE

FACULDADE
CIÊNCIAS DA SAÚDE
Departamento de
Ciências Médicas

A AMOSTRA DE FLUIDO ORAL COMO FERRAMENTA PARA A MONITORIZAÇÃO DE ANTIDEPRESSIVOS NO ÂMBITO CLÍNICO E FORENSE

Nº DE IDENTIFICAÇÃO DA AMOSTRA:

____

Avaliação do estado físico:

☐ Gravidez
☐ Stress físico
☐ Stress emocional
☐ Infeção
☐ Trauma

☐ Doenças pré-existentes:
- ☐ Doenças hepáticas
- ☐ Doenças cardíacas
- ☐ Doenças renais
- ☐ Doenças endócrinas
- ☐ Outras doenças de foro psiquiátrico

  Se sim, qual? ______________________

Efeitos secundários que observa:

☐ Sensação de confusão / Agitação
☐ Sedação / Astenia
☐ Hipotensão ortostática
☐ Efeitos anticolinérgicos (Exemplo: retenção urinária, aumento da pressão intraocular, xerostomia, obstipação, boca seca)
☐ Insónia
☐ Alterações gastro-intestinais (Exemplo: náuseas, vómitos, diarreia)
☐ Disfunção sexual

☐ Aumento da pressão arterial
☐ Cefaleias/ tonturas/ vertigens/ zumbidos
☐ Sudação
☐ Tremores
☐ Palpitações
☐ Aumento ou diminuição do apetite
☐ Aumento ou diminuição do peso significativos
☐ Outro(s): ______________________________

Desde que toma esta medicação modificou os seus hábitos alimentares e o consumo de bebidas alcoólicas?

☐ Sim ☐ Não

Toma algum suplemento ou produto natural (Exº chás (hipericão, cidreira, entre outros), suplementos vitamínicos ou outros) para além da medicação*?*

☐ Sim ☐ Não

☐ Se sim, indique qual ou quais: ____

Muito obrigado pela sua colaboração.

SAMPLE
